# Supplementary material for: Cell and circuit origins of fast network oscillations in the mammalian main olfactory bulb
Source: eLife. 2021 Oct 18;10:e74213. doi: 10.7554/eLife.74213 (PMC8553344; doi:10.7554/eLife.74213)
Supplement: Transparent reporting form [file elife-74213-transrepform1.pdf]

eLIFE
1st Floor
24 Hills Road
Cambridge CB2 1JP, UK
P 01223 855340
W elifesciences.org
T @elife

eLife's transparent reporting form

We encourage authors to provide detailed information *within their submission* to facilitate the interpretation and replication of experiments. Authors can upload supporting documentation to indicate the use of appropriate reporting guidelines for health-related research (see EQUATOR Network), life science research (see the BioSharing Information Resource), or the ARRIVE guidelines for reporting work involving animal research. Where applicable, authors should refer to any relevant reporting standards documents in this form.

If you have any questions, please consult our Journal Policies and/or contact us: editorial@elifesciences.org.

Sample-size estimation

- You should state whether an appropriate sample size was computed when the study was being designed
- You should state the statistical method of sample size computation and any required assumptions
- If no explicit power analysis was used, you should describe how you decided what sample (replicate) size (number) to use

Please outline where this information can be found within the submission (e.g., sections or figure legends), or explain why this information doesn't apply to your submission:

Concerning our analyses of spike-time synchrony, firing periodicity, and coincident synaptic inhibition among mitral cells (MCs) and tufted cells (TCs) of the main olfactory bulb (MOB), the following is included in the **Data analysis** subsection of the **Materials and methods** section (lines 777-782): "Given the lack of available data on TC synchrony and potential cell-type differences, no *a priori* power analyses were performed to determine target sample sizes. Experiments were instead designed to encompass a comparable number of pairs as several previous studies of spike-timing computation and coincident inhibition among MOB principal cells (e.g., Schoppa and Westbrook, 2001; 2002; Schoppa, 2006; Arevian et al., 2008; Giridhar et al., 2011; Schmidt and Strowbridge, 2014; Arnson and Strowbridge, 2017)."

Concerning our analyses of subthreshold oscillations among MCs and TCs, we elected to re-analyze a pre-existing dataset (Burton and Urban, 2014), which not only allowed us to reduce experimental animal use, but which also previously proved sufficient to resolve several cell-type differences among MCs and TCs, suggesting sufficient statistical power to uncover any additional differences in subthreshold oscillations.

Replicates

- You should report how often each experiment was performed
- You should include a definition of biological versus technical replication
- The data obtained should be provided and sufficient information should be provided to indicate the number of independent biological and/or technical replicates
- If you encountered any outliers, you should describe how these were handled
- Criteria for exclusion/inclusion of data should be clearly stated
- High-throughput sequence data should be uploaded before submission, with a private link for reviewers provided (these are available from both GEO and ArrayExpress)

Please outline where this information can be found within the submission (e.g., sections or figure legends), or explain why this information doesn't apply to your submission:

Biological replicates (i.e., cells/pairs recorded) are explicitly detailed within figures or, where results are stated without an accompanying figure, in the text of the **Results** section.

Experimental/technical replicates (i.e., trials recorded per cell/pair) are detailed in the **Results** section (lines 466-467) as well as the **Data analysis** subsection of the **Materials and methods** section (lines 792-795, 808, 810-811, 815-818).

Concerning outliers, the **Data analysis** subsection of the **Materials and Methods** section (lines 843-845) details that: "For visual comparisons of non-normally-distributed data, data are displayed as standard boxplots, with data points denoting sample outliers," with an outlier defined as a value more than 1.5× the interquartile range above or below the 75th or 25th percentile of the data, respectively (i.e., the standard settings of the Matlab boxplot function).

As specified in the **Data analysis** subsection of the **Materials and Methods** section (lines 794-795): "trials with spontaneous bursts of spikes or long-lasting depolarizations immediately preceding photostimulation onset were excluded from analysis." Otherwise, no data points (including data sample outliers in non-normally distributed data discussed above) were excluded from analysis.

Statistical reporting

- Statistical analysis methods should be described and justified
- Raw data should be presented in figures whenever informative to do so (typically when N per group is less than 10)
- For each experiment, you should identify the statistical tests used, exact values of N, definitions of center, methods of multiple test correction, and dispersion and precision measures (e.g., mean, median, SD, SEM, confidence intervals; and, for the major substantive results, a measure of effect size (e.g., Pearson's r, Cohen's d)
- Report exact p-values wherever possible alongside the summary statistics and 95% confidence intervals. These should be reported for all key questions and not only when the p-value is less than 0.05.

Please outline where this information can be found within the submission (e.g., sections or figure legends), or explain why this information doesn't apply to your submission:

Concerning the presentation of data and p-values, the **Data analysis** subsection of the **Materials and methods** section (lines 842-847) details that: "For visual comparison of normally-distributed data, all individual data points are displayed in addition to sample mean and standard errors. For visual comparison of non-normally-distributed data, data are displayed as standard boxplots, with data points denoting sample outliers. Values in text are reported as mean ± standard deviation. Line plots with shading denote mean ± standard error, except where noted. Single, double, and triple asterisks in figures denote statistical significance at $p<0.05$, $p<0.01$, and $p<0.001$ levels, respectively."

Concerning statistical analysis methods, the **Data analysis** subsection of the **Materials and methods** section (lines 841-842) details that: "For each statistical test, data normality was first determined by the Shapiro-Wilk test, and non-parametric tests applied where appropriate." Further, each statistical test applied is explicitly detailed in figure captions or, where results are stated without an accompanying figure, in the text of the **Results** section, with exact p-values given in all cases (including non-significant results) and effect sizes and degrees of freedom provided where appropriate (e.g., t-tests, F-tests, etc.).

(For large datasets, or papers with a very large number of statistical tests, you may upload a single table file with tests, Ns, etc., with reference to sections in the manuscript.)

eLife Sciences Publications, Ltd is a limited liability non-profit non-stock corporation incorporated in the State of Delaware, USA, with company number 5030732, and is registered in the UK with company number FC030576 and branch number BR015634 at the address 1st Floor, 24 Hills Road, Cambridge CB2 1JP | August 2014

Group allocation

- Indicate how samples were allocated into experimental groups (in the case of clinical studies, please specify allocation to treatment method); if randomization was used, please also state if restricted randomization was applied
- Indicate if masking was used during group allocation, data collection and/or data analysis

Please outline where this information can be found within the submission (e.g., sections or figure legends), or explain why this information doesn't apply to your submission:

Sample allocation (e.g., by cell type) is explicitly detailed throughout the **Results** section. No masking was possible in assigning cells/pairs to specific cell-type groups, as MCs and TCs were discriminated at the time of recording by anatomical differences (and confirmed with post-hoc visualization of Neurobiotin labeling). For assignment of data to other groups (e.g., resonant versus non-resonant cells), assignment was accomplished using automated code-based analyses, with distinguishing criteria explicitly detailed throughout the **Results**.

Additional data files ("source data")

- We encourage you to upload relevant additional data files, such as numerical data that are represented as a graph in a figure, or as a summary table
- Where provided, these should be in the most useful format, and they can be uploaded as "Source data" files linked to a main figure or table
- Include model definition files including the full list of parameters used
- Include code used for data analysis (e.g., R, MatLab)
- Avoid stating that data files are "available upon request"

Please indicate the figures or tables for which source data files have been provided:

Source data for Figures 1-9 and the associated figure supplements can be found in the Additional file "Burton and Urban, 2021 sourcedata.xlsx"
